# Jointly modelling longitudinally measured urinary human chorionic gonadotrophin and early pregnancy outcomes

**DOI:** 10.1038/s41598-020-61461-w

**Published:** 2020-03-12

**Authors:** N. B. Ashra, L. Marriott, S. Johnson, K. R. Abrams, M. J. Crowther

**Affiliations:** 1 Department of Health Sciences, University of Leicester, Leicester, UK; 2 Clinical Research Department, SPD Development Company Ltd., Bedford, UK

**Keywords:** Medical research, Biomarkers, Outcomes research

## Abstract

Human chorionic gonadotrophin (hCG) is largely used to confirm pregnancy. Yet evidence shows that longitudinal hCG profiles are distinguishable between healthy and failing pregnancies. We retrospectively fitted a joint longitudinal-survival model to data from 127 (85 healthy and 42 failing pregnancies) US women, aged 18–45, who were attempting to conceive, to quantify the association between longitudinally measured urinary hCG and early miscarriage. Using subject-specific predictions, obtained uniquely from the joint model, we investigated the plausibility of adaptively monitoring early pregnancy outcomes based on updating hCG measurements. Volunteers collected daily early morning urine samples for their menstrual cycle and up to 28 days post day of missed period. The longitudinal submodel for log hCG included a random intercept and slope and fixed linear and quadratic time terms. The survival submodel included maternal age and cycle length covariates. Unit increases in log hCG corresponded to a 63.9% (HR 0.36, 95% CI 0.16, 0.47) decrease in the risk of miscarriage, confirming a strong association between hCG and miscarriage. Outputted conditional survival probabilities gave individualised risk estimates for the early pregnancy outcomes in the short term. However, longer term monitoring would require a larger sample size and prospectively followed up data, focusing on emerging extensions to the joint model, which allow assessment of the specificity and sensitivity.

## Introduction

Early miscarriage, defined in the UK as loss before week 13, is a frequent complication of pregnancy^1^. It affects 12% to 24% of clinically confirmed pregnancies, not counting those losses which occur prior to the date of the missed period - so-called biochemical pregnancies^2^. Women who suffer from a miscarriage are more likely to report symptoms associated with depression, with affected women ranging from 20% to a high of 55%^3^. Though the majority of losses are self-resolving, those that are not may require diagnostic tests, hospital treatment, surgical intervention and follow-up care^2^. This provides an incentive to identify potential early losses as early as possible by exploring more patient-centred monitoring strategies.

The recently published priorities for research within miscarriage ranked highest the identification of effective interventions to prevent miscarriage^4^. This encompasses the plausibility of using biomarkers to track pregnancy progression through viability or miscarriage. Several potential biomarkers have been identified to predict miscarriage, with human chorionic gonadotrophin (hCG) a strong contender^5^. The hormone tends to rise rapidly and reliably in early pregnancy, doubling every 1.5 days in the first 5 weeks post conception and then every 3.5 days from week 7, before plateauing around week 10^5,6^. Its use is more prevalent in tracking early pregnancy progress in an *in vitro* fertilisation (IVF) population and for identifying ectopic pregnancies^7^. However, evidence suggests that longitudinal profiles of hCG can be utilised to distinguish between viable and failing pregnancies, with similar patterns of hCG noted across maternal serum and urine^8^.

The repeated collection of a continuous biomarker, such as hCG, over time gives rise to intermittently observed longitudinal data which are subject to measurement error^9,10^. Conventionally, this data is analysed using linear mixed effects models, with time-to-event outcomes analysed using survival models^11,12^. However, when interest lies in quantifying the association between the repeatedly measured biomarker and time-to-event outcome, separate analyses ignore the dependency between the longitudinal and time-to-event processes^13^.

Acknowledging an association between a longitudinal biomarker and survival outcome implies that very high or low values of the biomarker are indicative of adverse outcomes^14^. Fitting a simple survival model to the event, including all of the longitudinal biomarker information, tells us how a change in biomarker value affects survival over follow-up time. However, the variation in biomarker observations between individuals is not incorporated into the model, so inferences for individuals cannot be drawn. Secondly, the implicit changes in biomarker values between each physically observed measurement are ignored, resulting in a failure to build a complete biomarker profile. The linear mixed effects model can build this biomarker trajectory and inbuilt random effects allow estimation of personalised risk as an output of the model. Recognizing the advantages of both types of model, combining both the linear mixed effects and survival models through a shared dependence structure via the joint longitudinal-survival model is essential. This allows the association to be appropriately modelled, whilst taking into account the intermittent nature of observations and measurement error. The model, through estimation of individual trajectories, can aid monitoring and potentially prediction of outcomes.

The aim of this paper is to retrospectively apply the classical joint model framework to data of pregnant women, who were followed up from before conception, to quantify the association between longitudinal urinary hCG observations and early miscarriage. The paper will also consider whether estimation of conditional survival probabilities from the joint model could provide the basis for dynamic monitoring of patients in the very early stages of pregnancy prior to other symptoms manifesting.

## Results

A total of 44 (17.6%) women suffered miscarriages. The dataset used for analysis consists of 85 randomly selected viable pregnancies and 44 miscarried pregnancies. A summary of demographic variables is given in Table 1. Overall, the two groups were comparable. Women who experienced healthy pregnancies were slightly younger (mean ± SD: 29.95 ± 4.15) than those who miscarried (mean ± SD: 32.34 ± 4.60). The majority of women in either group were from a White European background (88.24% and 77.27% respectively). A slightly higher proportion of women who had viable pregnancies had previously experienced a miscarriage, compared to women who miscarried (12.94%, and 9.76%). Of the women who miscarried, 18 (14.2%) experienced biochemical pregnancies and 24 (57.1%) women suffered early miscarriages. Two women who miscarried did not contribute hCG measurements and were not included in the joint modelling analysis.

**Table 1:** Baseline demographics by pregnancy viability group.

Variables	Healthy (n = 85)	Miscarried (n = 44)	Overall (n = 129)
Age, years	29.95 (4.15)	32.34 (4.60)	30.77 (4.44)
**Ethnicity, n (%)**			
White	75 (88.24)	34 (77.27)	109 (84.50)
Black	3 (3.53)	7 (15.91)	10 (7.75)
Asian	4 (4.71)	2 (4.55)	6 (4.65)
Mixed	3 (3.53)	1 (2.27)	4 (3.10)
**Education, n (%)**			
High School	4 (4.71)	2 (4.55)	6 (4.65)
Graduate	69 (81.18)	28 (63.64)	97 (75.19)
Postgraduate	12 (14.12)	14 (31.82)	26 (20.16)
**Occupation, n (%)**			
Homemaker	12 (14.12)	3 (6.82)	15 (11.63)
Student	1 (1.18)	1 (2.27)	2 (1.55)
Skilled labourer	2 (2.35)	2 (4.55)	4 (3.10)
Office admin	8 (9.41)	5 (11.36)	13 (10.08)
Professional	60 (70.59)	31 (70.45)	91 (70.54)
Other	2 (2.35)	2 (4.55)	4 (3.10)
Cycle length	29.94 (2.95)	28.66 (3.21)	29.50 (3.09)
Previous pregnancies	1.00 (1.05)	1.11 (1.15)	1.04 (1.08)
Previous live births	0.62 (0.76)	0.70 (0.88)	0.65 (0.80)
Time to conceive, months	4.36 (5.83)	4.55 (5.98)	4.43 (5.86)
Previous miscarriage, n (%)	11 (12.94)	4 (9.76)	15 (11.90)

All values are mean(SD) unless otherwise stated.

The remaining 127 women all contributed repeated hCG measurements. For women who miscarried the average number of hCG observations was 17.5 (SD: 8.6) and for women who experienced viable pregnancies the average number of measurements was higher at 23.6 (SD 3.9).

**Figure 1:**
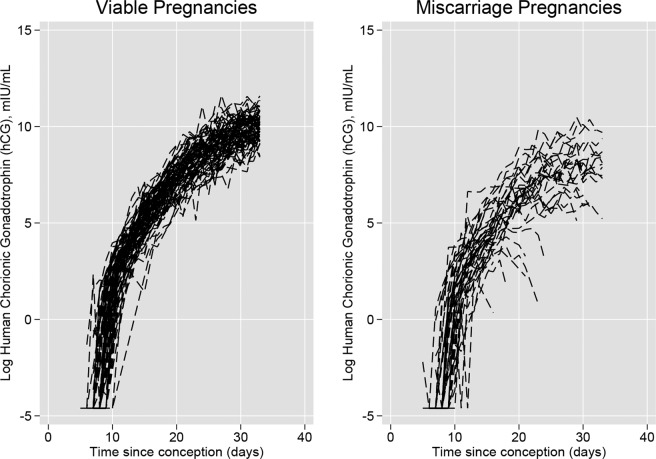
Log human chorionic gonadotrophin trajectories for viable pregnancies and miscarriage pregnancies.

Profiles of log hCG measurements for viable and failing pregnancies are presented in Fig. 1. The general trajectory shows an initial rise after conception, which continues through the first three weeks of the healthy pregnancies before slowing in rise. There was greater variation in profiles for women who miscarried, who also presented with an initial rise after conception. However, some women experienced a sharp drop in hCG, whilst others experienced a more gradual rise in hCG in comparison with women who had healthy pregnancies.

**Figure 2:**
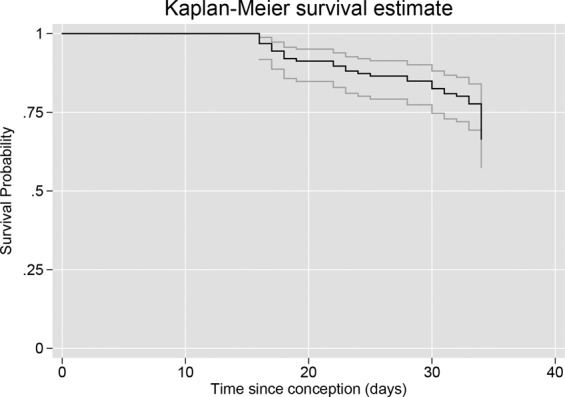
Kaplan-Meier survival probabilities for time-to-miscarriage.

Overall Kaplan-Meier survival estimates for time to miscarriage are shown in Fig. 2. An approximate 15 day lag is evident before an event is seen, due to the use of time since conception as a timeline.

### Modelling longitudinal profile

Inclusion of a quadratic time variable was necessary to appropriately capture the shape of the log hCG profile. Results from an initial fitted linear mixed effects model, including a grouping variable for pregnancy outcome, confirmed that mean log hCG was −1.66 mIU/mL (95% CI −2.14, −1.18) lower in the biochemical pregnancy group and −1.13 mIU/mL (95% CI −1.48, −0.78) lower in the early miscarriage group, when compared with the healthy pregnancies. Results are presented in Table 2.

**Table 2:** Model estimates from a linear mixed effects model.

Longitudinal model	Mean change in log hCG MIu/ml	95% Confidence Interval
Time since conception, days	1.431	1.396, 1.466
Quadratic time since conception, days	−0.025	−0.026, −0.024
**Group**		
Healthy	—	—
Biochemical loss	−1.656	−2.135, −1.176
Early loss	−1.132	−1.484, −0.781

### Joint longitudinal-survival model

A joint longitudinal-survival model was fitted to the data. Estimates for the model with current value association structure are given in Table 3. A unit increase in absolute value of log hCG corresponded to a 66.1% (HR 0.339, 95% CI 0.257, 0.447) decrease in the risk of miscarriage at time t. A one-year increase in maternal age at conception resulted in a 7.6% (HR 1.076, 95% CI 0.998, 1.159) increase in the risk of miscarriage. A one-day increase in cycle length was associated with a 15.6% (HR 0.844, 95% CI 0.739, 0.965) decrease in the risk of miscarriage.

**Table 3:** Model estimates from a joint longitudinal-survival model with current value association structure.

Survival submodel	Hazard Ratio	95% Confidence Interval
Age, years	1.076	0.998, 1.159
Usual cycle length, days	0.844	0.739, 0.965
Expected current value of log hCG	0.339	0.257, 0.447
**Longitudinal submodel**	**Mean**	**95% Confidence Interval**
Time since conception, days	1.431	1.396, 1.466
Quadratic time since conception, days	−0.025	−0.026, −0.024

A comparison of log hCG association parameters for various models are presented in Table 4. The association between log hCG and time to miscarriage was attenuated when fitting a survival model with time-varying covariate (HR: 0.439, 95% CI: 0.373, 0.516) and the two-stage model (HR: 0.440 95% CI: 0.368, 0.527). Furthermore, standard errors for both the standard survival model and two-stage model were 0.036 and 0.040 respectively compared to a larger 0.142 for the joint model.

**Table 4:** Survival estimates from a standard survival model with time-varying covariate, two-stage model and joint model.

Model	Standard error	Hazard Ratio for log hCG	95% Confidence Interval
Time-varying covariate	0.036	0.439	0.373, 0.516
Two-stage model	0.040	0.440	0.368, 0.527
Joint model	0.142	0.339	0.257, 0.447

### Conditional survival predictions

**Figure 3:**
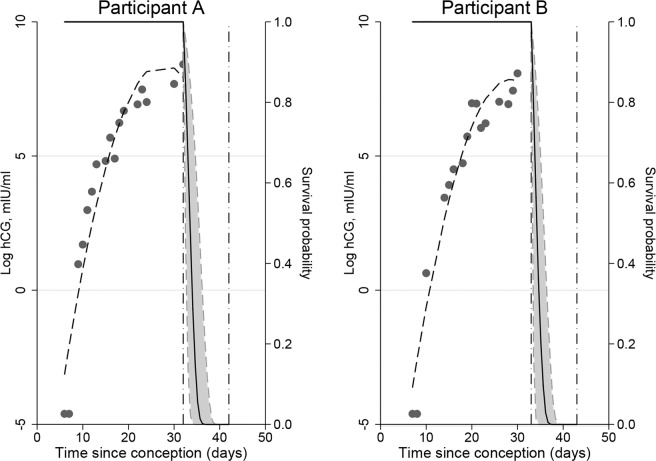
Conditional survival probability curves for participants A and B who experienced biochemical and early losses, respectively.

**Figure 4:**
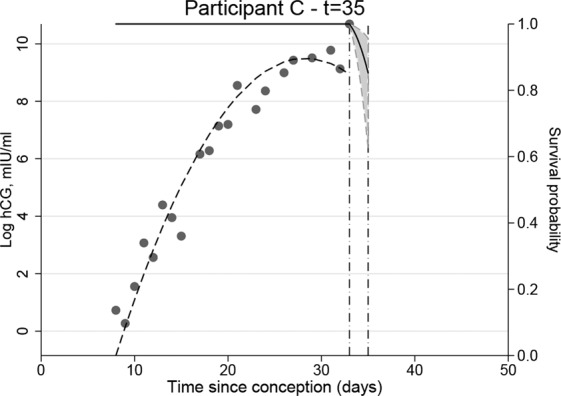
Conditional survival probability curve for participant C who experienced a healthy pregnancy.

Conditional survival probabilities were obtained from the joint model, which included the current value association structure (see Table 2). Probabilities estimated for the ten-day window after the last observed hCG measurement are shown in Fig. 3 and for a two-day window in Fig. 4. Participants A and B experienced biochemical and early losses respectively, whilst participant C experienced a healthy pregnancy. For both participants A and B a similar number of measurements were observed over comparable time periods, with similar average cycle lengths (28 and 30 days respectively) and ages (42 and 38 years respectively). Based on observed hCG measurements as well as age and cycle information, both were predicted to experience miscarriages.

For participant C estimates confirmed an 80% survival probability for the pregnancy two days post last observed hCG measurement. Depending on the cut-off used for low risk this may not be considered a high enough survival probability for a healthy pregnancy. As follow-up did not continue it was not possible to update probabilities to look at longer-term outcomes.

## Discussion

### Principal findings

This analysis builds on the two-stage model approach implemented by Marriott *et al*.^15^. By utilising the more advanced joint longitudinal-survival framework, the association between longitudinally measured urinary hCG and time to miscarriage is modelled, accounting for both measurement error and the intermittent nature of observations. This improves upon the two-stage model, which assumed that measurements remained constant between observation times.

With the emphasis now on personalised care, it is becoming standard practice to use the joint model in favour of singular or two-stage analyses to model the association between longitudinal and failure processes, to both maximise efficiency and minimise the potential for bias^16^. The mainstream use of joint models coincides with improvements in software making these complicated models increasingly easier to fit, with packages available in both R (JM, JoineRML) and Stata (stjm, merlin)^13,17–19^. This makes the estimation of conditional survival probabilities from such models more accessible.

This paper investigates whether urinary hCG could be used to monitor pregnancy viability prospectively in early pregnancy from first detection of hCG. Tracking at this early stage presents an adjunct to diagnosis by ultrasound later on in the pregnancy. This analysis echoes research suggesting declines in hCG can be noted even prior to other symptoms presenting^20^. There is also potential for this monitoring to occur prior to conception, with a recent study finding that a lag between the luteal phase and hCG production can be indicative of a biochemical pregnancy, possibly due to early or delayed implantation^21^.

Tracking of hCG by pregnant women is practicable, as demonstrated by Foo *et al*. who employed a fertility monitor that also provide semi-quantitative analysis of hCG levels on pregnancy tests that were used daily in women who conceived^21^. Retrospective analysis of the semi-quantitative data indicated that non-viable pregnancies had different hCG profiles to viable pregnancies. Serial tracking could have the potential to cause stress, although women using tests to track ovulation for fertility purposes do not appear to have higher stress levels than those not employing tests^22,23^. Nevertheless, it is likely that tracking would initially be of benefit in high risk pregnancies, where anxiety levels are already high and there would be a willingness and reason to track. Further research would be required to understand the psychological impact of tracking.

Monitoring from first detection has the potential to be useful in cases of recurrent miscarriage, particularly as research into treatment gains traction. A recently published feasibility study assessing the effectiveness of the diabetes drug sitagliptin as a treatment for recurrent miscarriage, presented promising findings^24^. This trial builds on previous research, which found that in some cases of recurrent miscarriage it is the deterioration of stem-like cells in the uterus which contribute to pregnancy loss. When adjusted for age and baseline colony forming unit (CFU) counts, the CFU count was higher (RR 1.52, 95% CI 1.32, 1.75) in the sitagliptin group compared to placebo, pointing to successful regeneration of cells. These findings could revolutionise treatment for unexplained recurrent miscarriage, particularly as the more established progesterone therapy has not been shown to significantly impact the rate of live births (PROMISE and PRISM trials)^25,26^.

Not all miscarriage is likely to be predictable due to the diverse aetiology of the condition. Some causes can be directly related to reduced hCG levels, e.g. conditions that affect rate of embryonic development such as chromosomal abnormalities, or inadequate placentation. Other causes, for example, where infectious agents or trauma are involved, may have no forewarning.

The demographic factors that add to the model have plausibility. The association between chronological age and miscarriage is well documented, and short follicular phase has also been associated with miscarriage by other authors^27,28^. Short cycle length may represent a surrogate marker for advanced reproductive age as the initial transition to peri-menopause can be characterised by a shortening of cycle length^29^.

### Strengths and limitations

The two-stage model did not allow the investigation of the nature of the association between miscarriage and hCG, something which is possible with the joint model. Although attempted it was not feasible to sensibly fit a joint model with a first derivative association structure, possibly due to the small sample size. This is something that requires further investigation in a larger dataset, particularly as there is evidence in the literature, which suggests that the overall profile of hCG is important as opposed to changes in absolute values of hCG. Certainly, both recent papers utilising Bayesian non-parametric models, and mixed effects penalized splines model approaches, focused on classification of each type of pregnancy based on complete longitudinal profiles^30,31^.

A Weibull model was utilised to model the baseline hazard, however ideally more flexibility would be desirable. This could be achieved by using restricted cubic splines to model the baseline hazard. Model selection was carried out using forwards stepwise selection, which is known to introduce bias^32^. Alternative selection methods should be considered in future, specific to the joint modelling context. Selection based on the log likelihood contribution for the longitudinal part and conditional survival model have been proposed, but are currently only implemented in the SAS statistical software^33^. The example dataset was relatively small, and so fitting a model as complex as the joint model was challenging. Results must therefore be interpreted with caution. As this was a retrospective analysis of data with limited follow-up measurements, it was not possible to update predictions as measurements were observed. Predictions were therefore inaccurate for wider time periods. With the small sample size, it was also not viable to split the dataset for development and validation of the model. Attempting to utilise such data for diagnostic or monitoring purposes also requires careful consideration of the potential for false positives. This study did not take into account the sensitivity and specificity of the fitted model, however this is an important component for planned future analyses in line with developments in joint model methodology^34,35^.

When utilising the joint model framework, it is essential to think about adjustments that need to be made to the model to truly reflect the biological reality of the biomarker and disease processes. In this analysis considerations were made for the timeline on which miscarriage was modelled and how this affected the inclusion of fixed and random effects. Due to limitations of the software, the models included fixed and random intercepts, though no hCG would have been detectable at time zero. Date of conception would also be unknown in a natural pregnancy setting, making this analysis more suited to an IVF setting. This, however, could be adjusted for by using the last menstrual period (LMP) as a timeline in a natural pregnancy setting.

Employing two separate modelling techniques for longitudinal and survival data requires larger sample size requirements in a clinical trial setting. The increased efficiency of simultaneously modelling the two outcomes has the advantage of maintaining desired power at a lower sample size^36^. This makes designing clinical trials around a joint model framework an attractive prospect.

## Conclusions

The novel extension to this analysis concerns the subject-specific predictions. This study is an initial investigation into whether women at high risk of miscarriage could be adaptively monitored via their urinary hCG concentration. Though the effectiveness of possible treatments, particularly for recurrent miscarriage, remain uncertain; the joint model is well placed for dynamic monitoring. However long term follow-up observations are required, along with access to a larger dataset for a model to be developed and subsequently validated. Future analyses should also consider the sensitivity and specificity of the fitted predictive model, to minimise the likelihood of false diagnoses of miscarriage.

## Methods

### Description of dataset

Women attempting to conceive were asked to collect daily early morning urine samples for their entire menstrual cycle and up to 28 days after the day of their missed period if they became pregnant. Women recruited were aged between 18–45 years and were not excluded on the basis of existing fertility issues. Intra-individual variation in the concentration of first morning urine is much lower, than when considering all urine voids. In addition, the exponential rise of hCG in early pregnancy from <1 mIU/ml to >150 000 mIU/ml renders fluctuations in urine concentration as having minimal effect on the trajectory of rise. Therefore, correction for urine concentration differences, e.g. using creatinine, was not required.

Urinary concentration of hCG was quantified using a validated quantitative immunoassay system (AutoDELFIA; PerkinElmer, Waltham, USA). The concentration of luteinising hormone (LH) in the urine were analysed by a panel of experts from a range of disciplines, including statisticians, endocrinologists and clinical scientists, to determine the day of LH surge, which occurs approximately 24 hours prior to ovulation. It was assumed conception occurred the day after the LH surge^37^. Details of sample collection and storage have been described previously^15^. Additional maternal demographics, menstrual and pregnancy history data were recorded. The study was carried out in accordance with the ethical principles of the Declaration of Helsinki. Written, informed consent was obtained from all individual participants involved in the study.

### Statistical analyses

The data utilised in this analysis have been analysed previously using a two-stage model approach^15^. The two-stage model utilises existing modelling techniques by first fitting a linear mixed effects model to the longitudinal data. Subject-specific predictions are then obtained from the mixed model and included as a time-varying covariate in a survival model. This method incorrectly assumes that the biomarker remains stagnant between measurements and gives too precise estimates with unrealistically small standard errors^16^. This analysis will now be extended using a joint model framework, which offers a number of advantages over the two-stage model approach.

### Longitudinal modelling

The joint model is made up of two component models – the longitudinal linear mixed effects model and proportional hazards survival model^36^. The longitudinal model for urinary log hCG forms a trajectory function, which estimates the unobserved values of log hCG for the ${i}^{th}\,\,$patient at time $t$ to form complete profiles. The formulation of the fitted model is as follows,

$$log\,hC{G}_{i}(t)={m}_{i}(t)+{e}_{ij}$$

$${m}_{i}(t)=({\beta }_{0}+{b}_{0i})+({\beta }_{1}+{b}_{1i})\,time+{\beta }_{2}\,tim{e}^{2}$$

$${b}_{i}\sim MVN(0,{\Omega }_{u})\,{e}_{ij}\sim N(0,{\sigma }_{e}^{2})$$

The model is made of fixed effects parameters including a fixed intercept ($${\beta }_{0}$$) and linear and quadratic time since conception terms, with parameter estimates, $${\beta }_{1}$$ and $${\beta }_{2}$$ respectively. The random effects parameters allow each individual $$i$$ to vary at baseline via a random intercept ($${b}_{oi})$$ and over time through a random linear time since conception term, with parameter estimate $${b}_{1i}$$. The possibility of measurement error, as with any continuous biomarker, is accounted for via the residual error term, $${e}_{ij},$$ which is normally distributed. The random effects $${b}_{i}$$ are multivariate normally distributed. An unstructured correlation matrix was assumed.

### Survival modelling

A proportional hazards survival submodel was assumed, conditional on ${M}_{i}(t)=\{{{\rm{m}}}_{{\rm{i}}}\,(s),\,0\le s\le t\}$, which denotes the history of the true unobserved longitudinal measurements up to time $t$ and additional covariates ${v}_{i}$^14^. The specific fitted model is given by,

$$h(t|{M}_{i}(t),{v}_{i})={h}_{0}(t)\exp [{\gamma }_{1}{\rm{age}}+{\gamma }_{2}\,usual\,cycle\,length+\alpha \,{m}_{i}(t)]$$

The baseline hazard, ${h}_{0}(t)$, was assumed to follow a Weibull distribution. Maternal age and usual cycle length were included as covariates in the survival model, after a forwards model selection procedure was carried out at the 5% significance level. The inclusion of ${m}_{i}(t)$ in the survival submodel estimates the change in absolute log hCG values and is termed the current value parameterisation, with association parameter $\alpha .\,\,$By including the longitudinal model within the survival submodel, we effectively link the expected value of log hCG to the miscarriage or censoring time, where typically an hCG response would not have been observed. Various association structures were explored, including the first derivative association structure, which models the rate of change of log hCG.

To allow for comparison a standard survival model with log hCG included as a time-varying covariate was fitted, as well as a two-stage model using subject specific predictions from the longitudinal model, as defined for the joint model, in a survival model.

Subject-specific survival probabilities dependent on maternal age and longitudinal log hCG measurements were obtained from the sample on which the joint model was fitted, using the Stata package stjm^13^. Conditional survival predictions can potentially be updated as measurements are observed, giving a real-time risk of miscarriage, or dynamic predictions. All models were fitted in Stata IC version 15.1.

### Funding and ethical approval

This was a diagnostic accuracy study on a sample bank collected from a multicentre, prospective study, conducted by Radiant Research (USA) on behalf of the sponsor SPD Development Company Ltd. (UK). The study was approved by Quorum Review Committee on 30^th^ November 2009; clinical trial number NCT01077583. This analysis was conducted by N.B.A as part of a doctoral training programme jointly funded by MRC IMPACT and SPD Development Company Ltd.

## Data Availability

The datasets analysed during the current study are not publicly available due to confidentiality restrictions in place between SPD Development Company Ltd. and the University of Leicester. Stata code written/used to perform the analysis are available from the corresponding author on reasonable request.
